# Factors associated with recidivism at a South African forensic psychiatric hospital

**DOI:** 10.4102/sajpsychiatry.v24i0.1125

**Published:** 2018-04-30

**Authors:** Nirvana Morgan, Giada Del Fabbro

**Affiliations:** 1 Department of Psychiatry, University of the Witwatersrand, South Africa; 2 Private Practice, South Africa

## Abstract

**Aims:**

This study examines common factors associated with recidivism among state patients at a South African forensic psychiatric hospital. More specifically, demographic, clinical and criminological factors of a recidivist group are compared to a non-recidivist group with the intention of understanding to what extent these factors might determine the likelihood of re-offending.

**Method:**

A retrospective case file review of 293 inpatients and a random selection of 120 outpatients was conducted. For the purpose of the study, a patient was classified as a recidivist if an additional charge or act of violence was added to the file while the patient was on leave of absence in the community. Of the inpatients, only those who met the criteria for recidivism were included in the study. All 120 randomly selected outpatients were included. Demographic, clinical and criminological data were captured for all patients.

**Results:**

Eighty recidivists were compared with 100 non-recidivists. Using the × 2 and Fischer’s exact test, substance-use disorder, antisocial personality disorder, an index offence of assault and in-ward adverse events were found to be associated with recidivism (*p* < 0.05). Using logistic regression analysis, the odds of recidivism in a patient with an index offence of assault was 8.4 times of those who did not commit assault as an index offence (95.0% CI 1.6–43.1). The odds of recidivism for patients with cannabis use was 2.8 (95.0% CI 1.3–6.0) and for patients with in-ward adverse sexual behaviour was 17.2 (95.0% CI 2.0–150).

**Conclusion:**

Substance-use disorder and antisocial personality disorder are associated with higher risk for recidivism. This study also highlights that a less serious offence such as assault had a higher association with recidivism. Patients noted to display adverse sexual behaviour in the ward pose a potentially high risk for re-offence. Important criminal history factors and certain clinical factors could not be interpreted because of large amounts of missing data in patients’ files.

## Introduction

The South African criminal justice system requires that all mentally disordered offenders (MDOs) who commit a serious or violent offence receive an evaluation by a forensic psychiatric unit. The 30-day assessment period is conducted by forensic psychiatrists and a multidisciplinary team. A report is compiled regarding the suspected MDO’s fitness to stand trial and his or her criminal capacity at the time of the alleged offence. In cases where the alleged perpetrator was determined to be unfit for trial and/or not criminally responsible because of the presence of a mental illness, the MDO is most likely to be declared a ‘state’ patient and diverted out of the criminal justice system to a forensic psychiatric facility for the purposes of care, treatment and rehabilitation.^1^ Within this system, forensic psychiatric teams are faced with the difficult task of risk assessment and managing repeat offenders.

Between 20.0% and 40.0% of persons with severe mental illness will encounter the criminal justice system at least once in their lifetime.^2^ It is important to consider how many of these MDOs will reencounter the criminal justice system because of recurrent offences, otherwise known as: recidivism. ‘A recidivist is one who after release from custody for having committed a crime […] falls back, or relapses into former behaviour patterns and commits additional crimes’.^3^

Measured rates of recidivism differ. A recent Swiss study found that 51.0% of MDOs were reconvicted, and of those, 13.0% committed violent re-offence.^4^ US studies demonstrate rates of re-offending of 30.0%^1^ and 70.0%^5^ and UK studies demonstrate rates of re-offending of 30.0%^6^ and 15.0%.^7^

The factors associated with recidivism among MDOs are generally divided into criminological, demographic and clinical factors. Criminological factors include variables such as age of first conviction, number of criminal charges and type of index offence. Criminological factors are most strongly associated with risk for recidivism. Younger age of first conviction and higher number of convictions have been the most reliable predictors of recidivism.^8,9,10,11,12^ Interestingly, these are the same risk factors as for the general offender. A relationship between the type of index offence and recidivism has also been noted. The Canadian National Trajectory Project^13^ found that MDOs with more serious index offences were less likely to reoffend, and similarly, in a Swiss study, it was found that MDOs with less serious index offences were more likely to reoffend.^4^ Although one may assume that an MDO with a more serious offence is more dangerous and more likely to reoffend, studies show that the relationship between index offence category and re-offence is far more complex.

With regard to demographic factors, many individual studies yield opposing results. The meta-analysis by Bonta et al.^14^ found that among demographic variables younger age, male gender and single marital status were predictors of recidivism. A second meta-analysis by Bonta et al.^8^ reported that problems with employment and being single were predictors of general and violent recidivism.

The two most important clinical variables linked to increased risk for recidivism are substance abuse and antisocial personality disorder.^8^ The presence of a mental illness alone, such as schizophrenia or bipolar disorder, has not been shown to be a significant predictor of recidivism.^6,12,15^ Coid et al.^16^ published the first study that compared multiple diagnostic subcategories and risk for re-offence. Patients with mania or hypomania were more than twice as likely to be reconvicted of a violent offence and four times more likely to commit a sexual offence. Some older studies have found lower level of intelligence to be predictive of recidivism^17,18^; however, once again, there are contrasting findings. A more recent study described that a lower level of intelligence was predictive of less crime.^19^ Length of stay in hospital is also an important clinical variable to consider as studies have repeatedly shown that a shorter length of stay in hospital was predictive of recidivism.^15,20^

A greater understanding of the patterns of recidivism within an MDO population may ultimately assist in more accurate risk assessment as well as a more individualised risk management intervention.^21^ Studies on recidivism in a forensic psychiatry context are confounded by different definitions of recidivism, different population samples and different judicial legislature regarding MDOs.^1^ This is the first South African study to assess factors associated with recidivism at a State South African forensic institute.

This study examines common factors associated with recidivism among state patients at a South African forensic psychiatric hospital. More specifically, demographic, clinical and criminological factors of a recidivist group are compared to a non-recidivist group with the intention of understanding to what extent these factors might determine the likelihood of re-offending.

## Methodology

The study design entailed a retrospective case file review.

### Study population

The study sample was drawn from the population of state patients at Sterkfontein Hospital. Both inpatient and outpatient files were selected for the review. Inpatients refer to patients that were admitted in the ward at the time of data collection and outpatients are patients that were on a leave of absence (LOA) at the time of data collection. Non-probability, purposive sampling was chosen for inpatients. Inpatients were included in the study only if they were found to be recidivists. This method was chosen to maximise the number of recidivists in the study and to minimise the logistical and ethical challenges regarding use of inpatient data. The control group (non-recidivists) was selected from the outpatient files. Outpatients’ files were chosen randomly.

### Inclusion criteria

- Adult (> 18 years) state patient of Sterkfontein Hospital.
- Re-offence that occurred in hospital was included in the recidivist group, provided a charge was laid.
- Clear documentation of criminal acts after becoming a state patient was deemed as a re-offence even in the absence of a charge.
- Patients that were recidivists and inpatients at the time of data collection were included provided that consent was obtained.
- No limitations were placed in terms of date of admission of patients.

For the purposes of this study, a recidivist was defined as one who reoffended or committed an act of violence after being declared a state patient. The recidivist category was divided into those against whom a formal charge was laid and those without a charge. To be classified a recidivist without a charge, the file had to contain a clear act of violence (or other serious offence) confirmed by collateral information while the patient was on LOA. We decided to include this category of recidivist into the sample as it is common for the South African Police Service to not charge a state patient when a crime is reported. A state patient is often escorted back to hospital rather than re-appearing in court.

### Exclusion criteria

- Female state patients. The study excluded females in order to create a more homogenous sample. It was also not an aim of the study to compare male and female state patients.
- Those who recidivated prior to becoming a state patient were not included in the recidivist group. Hence, recidivism was calculated from time of admission as a state patient. This decision was made based on lack availability of information in patient files.
- Inpatients were excluded from the sample unless the patient had reoffended and consent was obtained.
- Patients who were made involuntary mental health care users after their first charge and then a state patient on the second charge were not categorised as recidivists. Once again, this decision was made based on the limited information available in the clinical notes. Information gathered from the time of becoming a state patient was easier to obtain and thus more reliable.

A total of 293 inpatient files were reviewed. Sixty of these were classified as recidivists and included in the study. A random selection of 120 outpatient files were included for review. Data collection was completed between 2013 and 2014.

### Measures

The demographic variables assessed were:

- age at first admission
- highest level of education
- marital status
- employment status.

The clinical variables assessed were:

- DSM IV Axis I diagnosis
- DSM IV Axis II diagnosis (The DSM IV diagnoses were taken from the clinical file. The diagnoses were made by the multidisciplinary teams at Sterkfontein Hospital. Most of the clinical notes are written by psychiatry registrars).
- in-ward adverse events (This refers to behaviours in the ward which were not in keeping with ward rules and may cause harm to self, fellow patients or staff)
- duration of admission before first LOA
- time to recidivist offence (duration of time from becoming a state patient to re-offence).

The criminological variables assessed were:

- criminal history prior to becoming a state patient and number of charges
- category of index offence
- category of recidivist offence.

Patients with in-ward adverse events such as alleged adverse sexual behaviour or dangerous and aggressive behaviour in the ward were not included in the recidivist category unless a formal charge was laid. It can be argued that these are acts of violence and therefore should be counted as recidivist offences. The clinical files did not contain details of allegations made against the patients. It was also unclear if there was an investigation or a formal report into the incidents. Thus, the authors felt that automatically including these patients as recidivists could potentially confound the recidivist group.

### Statistical analysis

Comparison of the demographic, clinical and criminological characteristics of recidivists with and without a criminal charge was made using the *c*^2^ test. Fisher’s exact test was used for 2 × 2 tables or where the requirements for the *c*^2^ test could not be met. The demographic, clinical and criminological characteristics of the recidivist and non-recidivist groups were compared similarly.

Logistic regression analysis was used to examine the relative impact of age at first admission, relationship status, highest level of education, employment status, Axis I and II diagnosis, substance use, index offences and ward offences on the presence or absence recidivism (a binary dependent variable).

Given the large number of independent variables, and the sample size limitations, univariate logistic regression was first performed with each independent variable separately. Variables with a Wald statistic significant at *p* < 0.20 were retained for multivariate analysis. Before commencing multivariate analysis, bivariate association analysis was conducted among the independent variables: phi coefficients were determined between two dichotomous variables and Cramer’s V between two categorical variables. Several strong associations (phi coefficient or Cramer’s V in excess of 0.5) were identified, and hence, the variables retained for multivariate regression were grouped to avoid using strongly associated variables together. Twelve such variable groupings were analysed by multivariate logistic regression. Variables that were not significant at the 5.0% level were sequentially removed from the model. Many of the final models were identical once non-significant variables had been removed, and only three distinct models remained. Data analysis was carried out using SAS. The 5% significance level was used.

## Ethical consideration

The study was approved by the University of Witwatersrand Human Research Ethics Committee. Informed consent was obtained from patients who were inpatients at the time of data collection. Consent was obtained from family members in cases where the patient did not have capacity to consent.

## Results

**FIGURE 1:**
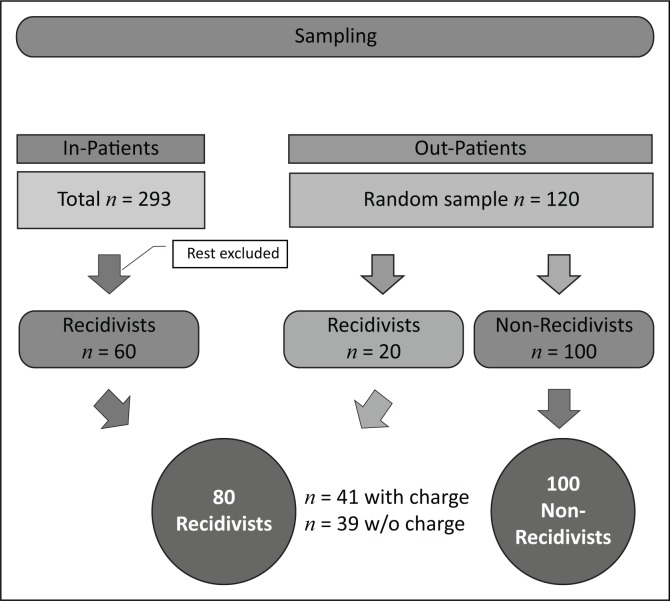
Sampling.

Of the 293 inpatient files reviewed, 60 were classified as recidivists. Out of the 120 outpatient files, 20 were recidivists. Thus, 80 recidivists were compared with 100 non-recidivists. Within the recidivist category, 41 had a formal charge laid and 39 were classified as recidivists without a charge. Statistical comparison was made between recidivists with a charge and recidivists without a charge (Figure 1). Notably when comparing those with a charge and those without a charge, there were no significant differences in any of the demographic, clinical or index offence variables. This further justified combining the two groups into one recidivist group for further analysis.

### Demographics

The average age of the patients was 30.7 years; 87.0% of the population were single and 7.0% were married. The patients were predominantly educated up to primary school level (32.0%). A fair proportion (31.0%) attended high school but did not complete their schooling.

When comparing recidivists (R) and non-recidivists (NR), there were no significant associations between age distribution (*p* = 0.88), marital status (*p* = 0.56), level of education (*p* = 0.51) and employment status (*p* = 1.00).

### Clinical factors

The most common non-substance-related diagnosis was schizophrenia (59.0%). Bipolar disorder and psychosis secondary to head injury both accounted for 8.7%. Schizoaffective disorder (SAD) accounted for 6.7% and 6.0% were diagnosed with psychosis secondary to epilepsy (Table 1).

**TABLE 1:** Clinical variables – Comparison between recidivists and non-recidivists.

Category	Overall (*n* = 180)		Non-recidivists (*n* = 100)		Recidivists (*n* = 80)		*p*
	*n*	%	*n*	%	*n*	%	
**Axis I diagnosis (*n* = 150)**							
Substance use	119	66.1	56	56.0	63	78.8	**0.0015**
Schizophrenia	88	58.7	48	60.8	40	56.3	0.62
Psychosis secondary to head injury	13	8.7	6	7.6	7	9.9	0.77
Bipolar disorder	13	8.7	7	8.9	6	8.5	1.00
SAD	10	6.7	2	2.5	8	11.3	0.047
Psychosis secondary to epilepsy	9	6.0	8	10.1	1	1.4	0.036
Psychosis NOS	7	4.7	4	5.1	3	4.2	1.00
SIPD	7	4.7	2	2.5	5	7.0	0.26
Dementia	3	2.0	2	2.5	1	1.4	1.00
Other	1	0.7	0	0.0	1	1.4	0.47
**Axis II diagnosis (*n* = 62)**							
Intellectual disability	46	74.2	32	100.0	14	46.7	**< 0.001**
ASPD	10	16.1	1	3.1	9	30.0	**0.0051**
Antisocial trait	8	12.9	0	0.0	8	26.7	**0.0017**
Ward adverse events	49	27.2	11	11.0	38	47.5	**< 0.0001**

Note: Significance set at *p* < 0.05 is denoted in bold.

SAD, schizoaffective disorder; NOS, not otherwise specified; SIPD, substance-induced psychotic disorder; ASPD, antisocial personality disorder.

Overall, 66.0% of the sample used one or more substances. The most common substance used was cannabis and the second most common was alcohol.

There were significant associations between recidivists (R) and non-recidivists (NR) for three Axis I conditions:

- A higher proportion of R were substance users compared to NR (*p* = 0.0015, phi coefficient = 0.24).
- A higher proportion of R had schizoaffective disorder (SAD) compared to NR (*p* = 0.047, phi coefficient = 0.17).
- A lower proportion of R had psychosis secondary to epilepsy compared to NR (*p* = 0.036, phi coefficient = 0.18).

Of the sample, 34% had a DSM IV Axis II diagnosis. Significant association was found between the three diagnostic categories and recidivism:

- A lower proportion of R had intellectual disability (ID) compared to NR (*p* < 0.0001; phi coefficient = 0.60).
- A higher proportion of patients with antisocial personality disorder (ASPD) and antisocial traits were recidivists (*p* = 0.0051 and *p* = 0.0017 respectively).

Overall, 27% of the patients were involved in in-ward adverse events. There was a significant association between in-ward adverse events and recidivism (*p* < 0.0001, phi coefficient = 0.41).

The most common adverse event was dangerous and aggressive behaviour (47.0%) followed by abscondment (43.0%) and adverse sexual behaviour (20.0%).

With regard to duration of admission before first LOA and time to recidivist offence, a large percentage of the data were missing from patients’ files and thus could not be analysed as predictors of recidivism. Time to recidivist offence measured the time interval between first admission as a state patient and the subsequent act of recidivism; 30.0% of the data were missing for this variable. Thus, data should be treated with caution. In 75.0% of cases in whom these data were available, the majority reoffended after 2 years.

### Criminological variables

The most common index offence was rape (34.4%) followed by assault with grievous bodily harm (GBH) (19.4%) and murder (16.7%); 6.7% of the sample committed assault, 6.1% committed indecent assault, 6.1% were charged with theft, 5.0% were charged with malicious damage to property (MDP) and 4.0% were charged with robbery with aggravating circumstances. Less than 4.0% of the sample had charges of attempted rape, attempted murder, arson, kidnapping or minor offences (Table 2).

**TABLE 2:** Criminal variables – Comparison between recidivists and non-recidivists.

Variable	Category	Overall (*n* = 180)		Non-recidivists (*n* = 100)		Recidivists (*n* = 80)		*p*
		*n*	%	*n*	%	*n*	%	
**Index offence: individual offence type**								
Murder		30	16.7	19	19,0	11	13.8	0.42
Attempted murder		3	1.7	2	2,0	1	1.3	1.00
Rape		62	34.4	35	35.0	27	33.8	0.88
Attempted rape		5	2.8	3	3.0	2	2.5	1.00
Indecent assault		11	6.1	6	6.0	5	6.3	1.00
Other sexual offence		1	0.6	1	1.0	0	0.0	1.00
Assault GBH		35	19.4	23	23.0	12	15.0	0.19
**Assault**		12	6.7	2	2.0	10	12.5	**0.006**
Robbery with aggravating circumstances		8	4.4	5	5.0	3	3.8	0.73
Armed robbery		0	0.0	0	0.0	0	0.0	-
Kidnapping		3	1.7	1	1.0	2	2.5	0.59
Arson		1	0.6	1	1.0	0	0.0	1.00
MDP		9	5.0	3	3.0	6	7.5	0.19
Housebreaking and robbery		8	4.4	3	3.0	5	6.3	0.47
Possession of unlicensed firearm		3	1.7	1	1.0	2	2.5	0.59
Theft		11	6.1	3	3.0	8	10.0	0.06
Minor offence		6	3.3	1	1.0	5	6.3	0.09

Note: Significance set at *p* < 0.05 is denoted in bold.

GBH, grievous bodily harm; MDP, malicious damage to property.

There was a significant association with R/NR for assault (*p* = 0.026; phi coefficient = 0.21): A *higher proportion of R committed assault as the index offence*, compared with NR.

Using the level 2 crime classification (Table 3), it is noted that 43% of the index offences were sexual offences. The most common offence type was contact crime (89.4%).

**TABLE 3:** The following crime classification was used for analysis.

Level 1	Level 2
Contact crime	Murder
Contact crime	Attempted murder
Contact crime	Sexual offence
Contact crime	Assault GBH
Contact crime	Common assault
Contact crime	Robbery with aggravating circumstances
Contact crime	Common robbery
Contact crime	Kidnapping
Contact related crime	Arson
Contact related crime	MDP
Property related crime	Housebreaking and robbery
Property related crime	Theft of motor vehicle
Crime detected – police action	Drug possession
Crime detected – police action	Illegal possession of a firearm
Other serious crime	Other theft
Other serious crime	Shoplifting
Minor crime†	Minor crime

GBH, grievous bodily harm; MDP, malicious damage to property.

† , minor offences; intimidation, crimen injuria, public drinking and domestic violence.

**FIGURE 2:**
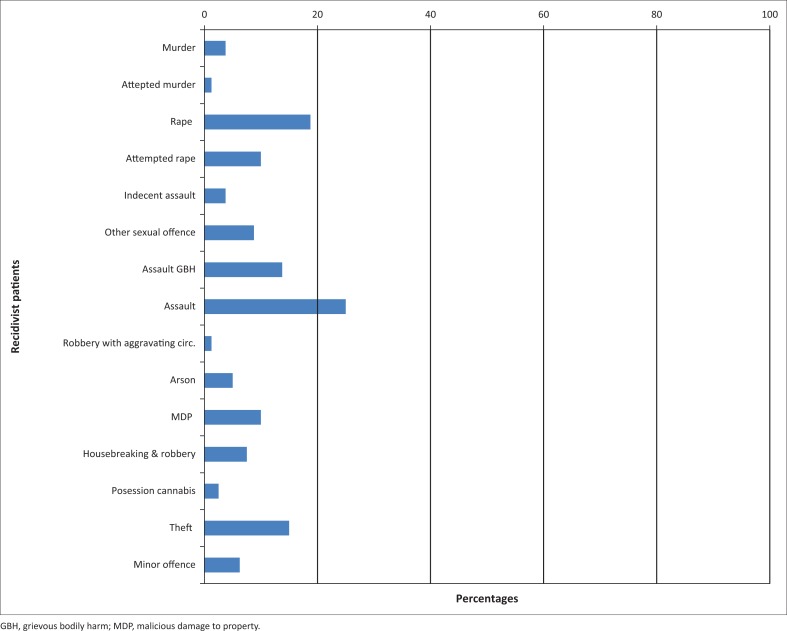
Percentage of recidivist patients (*n* = 80).

The most common recidivist offence was assault (25.0%) followed by rape (18.8%) (Figure 2). Using the level 2 classification, we find that 40.0% of recidivist offences were sexual offences.

Unfortunately, in 79.0% of patient files, data regarding history of criminal conviction prior to becoming a state patient and number of prior charges were absent.

The multivariate logistic regression analysis produced three models. One of these proved to be more clinically relevant and is therefore presented here (Table 4).

**TABLE 4:** Results of multiple logistic regression analysis.

Outcome: Recidivism	Phi-coefficient	Chi-square	*p*	OR (95% CI)
Cannabis only	−0.29	7.46	0.006	2.8 (1.3–6.0)
Polysubstance use	−0.07	5.58	0.018	4.4 (1.3–15.0)
Index offence – assault	−0.06	6.43	0.011	8.4 (1.6–43.1)
Absconded from ward	−0.16	4.48	0.034	3.3 (1.1–10.2)
In-ward adverse sexual behaviour	−0.04	6.63	0.010	17.2 (2.0–150.3)
In-ward aggressive behaviour	−0.08	8.79	0.003	5.3 (1.8–16.1)

OR, odds ratio; CI, confidence interval.

The odds of recidivism for those patients who used *cannabis (only)* was 2.8 times the odds of recidivism for those who did not use cannabis (OR = 2.8; 95.0% CI 1.3–6.0). The odds of recidivism for those patients with *polysubstance use* was 4.4 times the odds of recidivism for those without polysubstance use (OR = 4.4; 95.0% CI 1.3–15.0). The odds of recidivism for those patients with *assault as an index offence* was 8.4 times the odds of recidivism for those who did not commit assault as the index offence (OR = 8.4; 95.0% CI 1.6–43.1). The odds of recidivism for those patients who *absconded from the ward* was 3.3 times the odds of recidivism for those who did not commit this in-ward adverse events (OR = 3.3; 95.0% CI 1.1–10.2). The odds of recidivism for those patients who displayed *adverse sexual behaviour* in the ward was 17.2 times the odds of recidivism for those who did not commit in-ward adverse events (OR = 17.2; 95.0% CI 2.0–150).

## Discussion

### Demographic variables

The majority of the sample were single, were unemployed and did not complete schooling. These findings are in keeping with most other South African studies on forensic psychiatric patients.^22,23,24^ There were no significant differences in demographics between recidivists and non-recidivists. Bonta et al.^14^ did not find level of education to be a predictor of general or violent readmission. Phillips et al.,^15^ Friendship et al.^25^ and Edwards et al.^26^ did not find marital status to be a predictor of recidivism. Therefore, some results may be in keeping with previous studies. However, younger age of index offence has repeatedly been associated with recidivism.^8,14^ Our results are therefore unexpected. Possible reasons for this may be that age at *first admission* and not age at *first offence* was captured. This is largely because age at first offence is not easily available in the files. Furthermore, it should be noted that the study compares two clinical population groups and not MDOs to general offenders. This factor may also contribute to differing results.

### Clinical variables

Psychotic disorders comprised the majority of DSM IV Axis I disorders. International studies report a much higher rate of mood disorders than our study.^6,27^ Not a single patient in the sample was diagnosed with a major depressive disorder. This raises questions about missed diagnoses, different presentations of mood in different cultural contexts, low referral of depressed patients from the courts or a significant low prevalence of depression among Sterkfontein Hospital male state patients. Further research is required to explore this trend.

Schizoaffective disorder was positively associated with recidivism (*p* = 0.047). This result should be interpreted with circumspection as there were only 10 patients with this diagnosis. Coid et al.^16^ was the first study to assess individual diagnostic categories and recidivism. The aforementioned study did not report a similar association nor did it report a negative association between psychosis secondary to head injury and recidivism. The findings of our study may be unique to a South African context or may possibly be replicated in further international studies.

Overall, 66.0% of the sample used one or more substances. Cannabis and alcohol are repeatedly found as the most common substances used among state patients and the general South African population.^23,28^ However, previous studies on South African state patients report alcohol use as more prevalent than cannabis use. A possible but untested reason for this study’s finding may be a change in trend in substance use among MDOs in more recent years. The South African Community Epidemiology Network on Drug Use (SACENDU) also reported that in Gauteng substance rehabilitation facilities, cannabis use is more common than alcohol use.^29^ Substance-use disorders were significantly higher among recidivists confirming that substance use is a risk factor for recidivism. Substance-use disorders have been reported as one of the strongest predictors of recidivism in most studies in the field.^27^

Approximately 35.0% of the sample had an Axis II diagnosis. The most common diagnosis was ID (75.0%). Only 10 patients out of the total had a diagnosis of ASPD and 8 patients were reported to have antisocial traits. This finding is not in keeping with international studies. Most South African studies profiling state patients have not investigated or reported on ASPD. In North American studies, the frequency of personality disorders, especially ASPD, was 46.0% – 88.0%, and in European studies, it was 37.0% – 56.0%.^30^ In this study, it is not known whether the low percentage of personality disorders is because of a missed diagnosis of ASPD, poor documentation of patient diagnoses or a significantly different profile of South African MDOs. As was expected, there was a positive correlation between a diagnosis of antisocial personality or antisocial traits and recidivism demonstrating that this diagnosis may be a risk for re-offence. This is in keeping with the majority of studies in the field.^13^

A much lower proportion of recidivists were diagnosed with ID compared to non-recidivists (*p* < 0.05). Thus, the findings suggest that lower intelligence level is associated with less re-offence. It may, however, also be possible that people with IDs remain inpatients for longer durations and are therefore less likely to reoffend. This trend should be explored in future studies.

In-ward adverse events were much higher among the recidivist population. This suggests that patients that demonstrate violence in the ward may be more likely to reoffend. Using logistic regression, in-ward sexual events, such as sodomy, were found to be predictive of recidivism. Ward incidents may also relate to psychiatric symptom control or response to treatment. Therefore, this finding may suggest a more complex association between ward adverse events and psychopathology, which is then associated with criminal behaviour.

### Criminological variables

Almost 90.0% of index offences were violent crimes against a person (contact crime). Sexual offences accounted for 43.0% of index offences. Of the sexual offences, 34.4% were rape charges. The majority of the other contact crimes were assault GBH (19.4%) and murder (16.7%). These findings are in keeping with other South African studies.^22,24^ In comparison with international literature, however, violent crime against a person is much higher in South Africa. The results among state patients of Sterkfontein Hospital are in keeping with the national crime statistics.^31^ This trend may also reflect patterns of referral for observations.

An index charge of assault had a significant correlation with recidivism suggesting that those charged with assault initially were more likely to reoffend. A study on mentally ill offenders in Switzerland found that MDOs that committed less serious index offences, such as violation of narcotics law, property crimes, assault, and robbery, were closely associated with an elevated risk for re-offending.^4^ The Canadian National Trajectory Project reported a similar trend.^13^ Thus, our findings are in keeping with both Canadian and Swiss studies. As the association between an index charge of assault and re-offence has been found in two separate studies, it may be useful to explore this trend further in a larger sample and at another South African forensic psychiatric institute.

Of the total sample of 180 state patients, 80 were recidivists. It is not within the objectives of this study to assess the rate of recidivism as there was a sampling bias in favour of recidivists. Seventy-eight per cent of recidivist offences were violent crimes against a person. Of these, 40.0% were sexual offences and 25.0% were cases of assault. An American study by Lovell et al.^5^ reported that 72.0% of re-offences were minor crimes and serious re-offences occurred in only 4.4% of the MDOs. Friendship et al.^25^ reported that 17.8% of the re-offences were sexual offences and about 60.0% were violent (including murder, attempted murder, wounding and assault). It is therefore notable that findings regarding the nature of re-offence differ considerably.

Other criminological variables (charges prior to becoming a state patient, number of charges) and other clinical variables (time from admission to first LOA and time to recidivist offence) are important variables to consider when assessing risk. Unfortunately, because of limited information in patients’ files at Sterkfontein Hospital, these data were not available.

## Limitations

As mentioned above, the major limitation of the study was missing data. This is a common challenge with a retrospective approach. The absence of a digital database meant identifying recidivists was solely based on analysis of doctors’ notes.

The recidivist category included patients that had a charge laid against them and those that had documentation of violent acts even in the absence of a charge. It could be argued that including patients that did not have a formal charge into the ‘recidivist’ category impacts the reliability and validity of the study. To some extent, this may be a justifiable contention. When assessing the literature on violence, mental illness and recidivism, it is worth noting that the definition of re-offence or acts of violence differs considerably. In a South African context, the researchers felt it was justifiable to include patients that had a well-documented history of violent behaviour without a charge into the recidivist category as statistical analysis comparing patients with a charge to those without a charge showed no significant differences.

This study included patients from both inpatient and outpatient samples. It may have been ideal to restrict the study to only outpatients; however, the study required a sufficient number of recidivists. There were more recidivists within the inpatient population, and thus, these patients were included.

Potential recidivists may have been missed during the retrospective review as often information regarding patients’ violent or aggressive behaviour while in the community lacked detail with regard to the degree of violence or impact on the alleged victim. When information was not clearly documented or vague, patients were not included in the recidivist category. It is also possible that recidivists were not accounted for as information about what occurred in the community was unavailable to clinical staff and therefore not documented. A prospective approach to such a study is likely to overcome such challenges.

Another possible limitation is with regard to the control or ‘non-recidivist’ group. The control group consisted of outpatients only. These patients had been in the community for varying periods of time ranging from 6 months to over 5 years. For those in the community for a shorter period of time, it is not known whether they may still go on to reoffend. Thus, one could argue that the control group is not a true control. Literature in the field of recidivism show that re-offence occurs within the first 3 years of release.^27,32^ It was therefore difficult to decide if the non-recidivists had to have spent a specific duration in the community before including them into the control group. This study showed that the majority of patients reoffended after 2 years, which suggests that it may have been beneficial to limit the control group to those who were in the community for at least 2 years. More South African studies that are prospective in nature with large sample sizes and long follow-up periods are needed to accurately assess when state patients are at highest risk for re-offence.

This study only assessed three main categories of factors associated with recidivism – demographic, clinical and criminological. There may, however, be many other factors associated with recidivism that were not within the scope of the study. For example, sociological variables such as housing, family structure, family supervision and access to drugs may impact recidivism. Other clinical variables such as medication adherence were not assessed but have been shown to decrease violence.^33^ This study investigated issues from the perspective of forensic psychiatry rather than a psychological, social or law viewpoint. In a complex field such as forensic psychiatry and recidivism, it is, however, worthwhile combining and sharing information from different fields to adequately address the issue.^34^

The sample size (*n* = 180) was adequate for the logistic regression and most of the chi tests; however, the associations between certain variables and recidivism could have been strengthened by a larger sample. The sample was also limited to male state patients at Sterkfontein Hospital and thus may not be generalisable to the entire country. It would therefore be valuable to replicate this study in other forensic psychiatric institutes within South Africa in order to compare the findings.

## Conclusion

Substance-use disorder and ASPD are associated with higher risk for recidivism. This study also highlights that a less serious offence such as assault had a higher association with recidivism and that patients noted to display adverse sexual behaviour in the ward pose a potentially high risk for re-offence.

This is one of the first studies focusing on recidivism in a South African state patient population. Some of the results of the studies are in keeping with international literature; however, many factors found to be associated with recidivism are new and deserve further exploration. This study also highlighted some of the gaps in forensic psychiatry at Sterkfontein Hospital. Post-Apartheid South Africa has instituted mental health care legislature that aims to provide optimal treatment for MDOs and justice for society. However, more work needs to be conducted to ensure systems are in place to effectively implement this legislature.
